# Trapped in a Daydream: Daily Elevations in Maladaptive Daydreaming Are Associated With Daily Psychopathological Symptoms

**DOI:** 10.3389/fpsyt.2018.00194

**Published:** 2018-05-15

**Authors:** Nirit Soffer-Dudek, Eli Somer

**Affiliations:** ^1^The Consciousness and Psychopathology Laboratory, Department of Psychology, Ben-Gurion University of the Negev, Beer-Sheva, Israel; ^2^School of Social Work, University of Haifa, Haifa, Israel

**Keywords:** daydreaming, obsessive-compulsive disorder, dissociation, distress, fantasy, fantasy proneness, absorption, mind-wandering

## Abstract

**Background:** Maladaptive Daydreaming (MD) characterizes individuals who engage in vivid, fanciful daydreaming for hours on end, neglecting real-life relationships and responsibilities, resulting in clinical distress and functional impairment. Sufferers have embraced the term MD in cyber-communities devoted to this problem because it seemed to uniquely fit their experience and since existing diagnostic labels and their therapies seemed inadequate. However, scientific research in the field has been scarce, relying on cross-sectional or case study designs. Existing knowledge on MD suggests the involvement of dissociative and obsessive-compulsive symptoms, as well as positive reinforcement comparable to processes in addiction disorders. The present study aimed to rigorously explore factors that accompany MD employing a longitudinal daily-diary design, hypothesizing that temporal increases in MD will associate concurrently with, and will temporally precede, other symptoms and emotional changes. In addition, we aimed to explore which symptoms may act as precursors to increases in MD, in order to identify possible mechanisms bringing about daydreaming in these individuals.

**Methods:** In a sample of 77 self-diagnosed individuals with MD we assessed relevant daily symptoms for 14 days, including MD, depression, general anxiety, social anxiety, obsessive-compulsive symptoms, and dissociation, as well as positive and negative emotion.

**Results:** Increases in MD were strongly related to concurrent increases in all other symptoms and negative emotion, and to decreased positive emotion. Obsessive-compulsive symptoms, dissociation, and negative emotion also temporally followed MD. Obsessive-compulsive symptoms were the only consistent temporal antecedent of MD.

**Conclusions:** MD and obsessive-compulsive symptoms coincided in what seems to be a vicious cycle; understanding possible shared mechanisms between these symptoms may inform our understanding of the etiology of MD. For example, Serotonin levels may possibly be involved in the development or maintenance of this condition. The findings may also provide clues as to potentially beneficial interventions for treating MD. For example, perhaps utilizing response prevention techniques may be useful for curbing or intercepting unwanted daydreaming. Future studies on MD should address its compulsory nature.

## Introduction

“*I have been lost in daydreams for as long as I can remember […] Some daydreams involve people I know […] Others don't include me at all […] These daydreams tend to be stories–[…] for which I feel real emotion, usually happiness or sadness, which have the ability to make me laugh and cry […] They're as important a part of my life as anything else; I can spend hours alone with my daydreams […] I often feel as if I just cannot turn off my mind, whether because I need to concentrate in class, go to sleep, or just find some peace in the world outside my head […] Running, walking, and driving are more effective at invoking daydreams than sitting or lying still, and I sometimes pace the floor of my room while I daydream. These actions are not involuntary; I know I am doing them, and I can stop doing them at will, but there is definitely a connection between my daydreams and my physical movements […] I am careful to control my actions in public so it is not evident that my mind is constantly spinning these stories and I am constantly lost in them […] I can sustain normal relationships with friends, coworkers, and family, although I often neglect those relationships in favor of replaying or elaborating on my daydreams […] I am torn between the love of my daydreams and the desire to be normal”*

[20 year-old self-diagnosed female student from the United States, who approached the second author (ES) by email, describing what she believes to be her abnormal daydreaming behavior; reproduced with permission].

The current study aims to broaden the understanding of the nature of a recently identified disorder—not yet included in standard mental health diagnostic manuals—labeled Daydreaming Disorder (1) or as it is more commonly known, Maladaptive Daydreaming [MD; (2)], by exploring its daily associations with relevant psychopathological symptoms as well as negative and positive emotion. The cross-sectional nature of the existing research on MD makes it difficult to pinpoint key elements affecting symptom severity. By identifying co-occurring, precipitating, and outcome factors, we may better conceptualize the mechanisms and dynamics characterizing the daily lives of those suffering from MD.

Daydreaming is a widespread, normal mental activity experienced by almost everyone (3, 4). A rigorous experience-sampling study on a large non-clinical sample revealed that our minds wander from what we are doing in the present in almost half of our waking thoughts (5). Daydreaming or mind-wandering, i.e., engaging in self-generated thought which is discrepant from our present activity, probably has evolutionary benefits; for example, it may be important for prospection such as future planning or simulation, mental breaks to relieve boredom, engendering creativity, and finding meaning in one's personal experiences or creating one's life narrative (6–8). It is not entirely clear to what extent daydreaming or mind-wandering from a present task is deliberate, i.e., characterized by conscious control and awareness. Although straying from task-relevant to task irrelevant thought may represent a failure of executive control (9), it has also been claimed that once mind-wandering has been initiated, executive control is needed to ensure the continuity of a self-generated internal “train of thought” (10). Yet another conceptualization for mind-wandering is the breakdown of meta-awareness (11), including a reduction in one's ability to regulate consciousness back to a goal-directed state. Interestingly, mind-wandering without awareness to one's state is associated with greater psychopathology and disruption to functioning (8).

Perhaps due to the commonness of daydreaming, its relationships with psychopathological distress have hardly been investigated. Possibly, it has been assumed that if the phenomenon is normal, with adaptive functions, and almost everyone engages in it, it must have no relevance to psychopathology. However, the same could be argued about several phenomena which underlie well-established diagnostic disorders, such as sadness or apprehension. Although everyone experiences such feelings occasionally, and they certainly possess adaptive functionality, some people may feel extremely so, in a way that instigates clinically significant distress and functioning impairment (possibly due to a combination of biological and psychological etiological factors). So, can daydreaming also go awry?

Singer and Rowe (12) demonstrated decades ago that the frequency of daydreaming is associated with several anxiety measures. Later on, there were attempts to decipher which daydreaming styles are related to psychopathology (13–15), supporting the idea that daydreaming may be associated with guilt, dysphoria, and lack of attentional control. Constructs closely related to daydreaming have also demonstrated robust relationships with psychopathological symptoms; for example, “absorption and imaginative involvement,” a dissociative tendency for immersed awareness and involvement in fantasy [e.g., (16, 17)]. Absorption loads on a negative emotionality factor and associates with anxiety sensitivity and panic attacks (18). Directionality of the relation was demonstrated more recently by studies employing multi-wave designs and time-lag analyses; for example, mind-wandering is temporally followed by a decrease in happiness levels (5), possibly because when thoughts are focused on present actions they can promote well-being, as opposed, for example, to thinking about one's to-do list for the day or ruminating about yesterday's events. Similarly, another time-lag study found that dissociative absorption is temporally followed by an increase in obsessive-compulsive symptoms (19), possibly because coming out of an absorbed state of mind brings about anxiety and uncertainty regarding the events that have transpired, as well as subsequent checking behaviors meant to substantiate reality. Another altered-consciousness personality trait representing the tendency for immersion in one's imagination is fantasy proneness (FP) introduced by Wilson and Barber (20); indeed, it has also been found to represent a risk factor for major psychopathology (21). Klinger (22) suggested that the relationship between FP and psychopathology was because the FP assessment measure is confounded, rather than because of daydreaming being a manifestation of psychopathology^1^. Truly, it is important to differentiate normal from abnormal daydreaming; this is perhaps the major limitation of the studies mentioned above, which have mostly treated the constructs as continuous rather than considering abnormal daydreaming as a discrete phenomenon.

In recent years it has gradually become evident that daydreaming can evolve into an extreme and maladaptive behavior, up to the point where it turns into a clinically significant condition. MD is an immersive and addictive imagination activity that leads to distress because it hinders social, occupational, and academic performance (2). Suggested criteria for a diagnosis of MD (1) specify that the excessive daydreaming causes clinically significant distress or functional impairment, thus treating the “continuous vs. discrete” problem in the same way as does the framework of the Diagnostic and Statistical Manual of Mental Disorders, fifth edition [DSM-5; (25)]. Individuals with MD feel the need to engage in vivid, fanciful imagery that may last for hours on end. Some report that their daydreams involve compensatory narratives featuring idealized versions of themselves, while others report immersive soap-like plots which they “watch” in their minds, with characters aging appropriately over the years [(2), a qualitative study on 6 cases; (26), an open-ended survey of 90 self-identified maladaptive daydreamers; (27), a qualitative analysis of in-depth interviews with 21 respondents who identified their daydreaming as matching a provided description of MD]. Although scientific investigations on the subject have been sparse, thousands of Internet users have embraced the term MD; several cyber communities are devoted to individuals who suffer from MD and seek online communication with others who understand and share their condition. Many of these web surfers report that they have finally found a fitting description of their symptoms (e.g., the Yahoo Maladaptive Daydreamers forum^2^ with over 3,500 users, and the MD community in Wild Minds Network^3^ serving over 10,000 participants). While these figures are impressive and attest to the appeal of the MD concept to many, such anecdotal information is not a reliable enough source of information in order to establish our understanding of MD, attesting to the need for rigorous scientific research. Initial data collected from members of such communities indicated that participants had experienced distress stemming from three factors: difficulty in controlling the need or desire to engage in fantasizing; interference of the quantity of fantasizing with actual relationships and endeavors; and intense shame and exhaustive efforts to keep this behavior hidden from others, including mental health practitioners (26). Despite evidence that MD was associated with considerable suffering and dysfunctionality [(28), a comparison of individuals who were self-classified based on provided criteria as either maladaptive (*n* = 340) or non-maladaptive (*n* = 107) daydreamers], therapists were reported to often be dismissive of the condition, offer no help, or provide unsuccessful treatment for better known diagnoses [(26, 27), and see also (29), a clinical case study of a person diagnosed with both a validated MD scale and a reliable MD diagnostic interview]. A recent study on clinical-level maladaptive daydreamers, showed with a structured clinical interview that the sample was highly psychopathological; most had at least four additional diagnoses [(30), diagnostic results of structured clinical interviews with (31) individuals who met criteria for MD].

In addition to MD's sheer intensity and quantity, that bring about distress in these individuals, there are also some unique features which characterize MD and differentiate it from normal daydreaming or mind-wandering, and from related constructs such as FP. As opposed to normal daydreaming which is usually neither very immersive nor fanciful (31), the quality of daydreaming in MD seems to represent an innate talent for vivid fantasy (27). Fantasy, defined as “a fictional tale created by a subject for his own pleasure and for no other purpose” [(32), p. 6], is considered to occur much less frequently than daydreaming (3). However, MD is also different than FP. Although FP represents a category of persons who have highly vivid daydreams in which they engage for 50% of their waking hours, additional central features of FP are beliefs in parapsychological phenomena and confusion between fantasy and reality (33, 34); these features are absent in MD (26). Finally, a central feature of MD which differentiates it from both normal daydreaming and FP is a need for some additional stimulation; specifically, repetitive, or stereotypical kinesthetic activity and exposure to evocative music are usually important conditions for the facilitation of this mental activity [(2, 26)(35), a qualitative analysis of in-depth interviews of 16 individuals who identified themselves as meeting an elaborate description of MD]. These initial findings suggest that the phenomenon of MD is indeed a unique entity. The development of this embryonic research field may be invaluable in order to aid individuals with MD in clinical practice.

Further evidence for the utility of the MD construct was provided following the development of the Maladaptive Daydreaming Scale (MDS). The MDS demonstrated good face, convergent, and divergent validity with excellent sensitivity and specificity (36). A structured clinical interview was recently developed based on proposed diagnostic criteria for MD [SCIMD; (1)]. Based on the standards adopted for the fifth edition of the Diagnostic and Statistical Manual of mental disorders [DSM-5; (25)] field trials (37), the SCIMD was able to diagnose MD with very good reliability, providing further evidence for the usefulness of this proposed diagnostic nosology.

After establishing the validity and reliability of MD as a unique construct (1, 26, 36), what is currently lacking is an ontological conceptualization of what MD is, as well as an understanding of the possible pathways that may lead to the development of this disorder, in order to conceive of potentially useful interventions. It has been suggested (20) that MD may be a dissociative disorder, a disturbance of attention, a behavioral addiction, or an obsessive-compulsive spectrum disorder. Relating to the first possibility, although phenomenological descriptions of MD (27) and the suggested diagnostic criteria of the condition (1) include symptoms that are pathognomonic to MD and different than the characteristics of existing dissociative disorders, MD does indeed seem to contain several dissociative elements. Specifically: (a) detachment from external reality in favor of internal experience; (b) absorption—a state of total attention; and (c) via their daydreams, individuals may temporarily adopt alternative (non-self) identities (while acting out characters' behaviors or dialogues in their minds). Additionally, some individuals have described the initiation of excessive daydreaming during childhood to avoid an intimidating or traumatic social environment (2, 35). In other words, individuals suffering from an abusive environment or those who suffer from social anxiety disorder may develop MD as a means for escaping from the harsh reality into their safe internal worlds. Indeed, one study found that social anxiety and childhood trauma were correlated with MD (38). Such findings may point to a stress-diathesis model for MD, whereby individuals who have an innate talent for immersive and fanciful imagery may develop MD if they are burdened with stressful life events. However, individuals may also suffer from MD that is not instigated by any apparent clinical psychiatric disorder or childhood adversity; individuals often report that their daydreaming has addictive or compulsory qualities. For example, a case study of a patient with MD described excessive, addictive daydreaming that caused, but did not seem to be caused by, distress (39). The patient was successfully treated for over 10 years with fluvoxamine, that reportedly helped to control her daydreaming. The fact that this patient responded to a medication that influences serotonergic tone, implies neurochemical irregularity and suggests a potential association between MD and obsessive-compulsive spectrum disorders (29). Indeed, individuals with MD are higher in obsessive-compulsive symptoms, as well as in dissociation, compared to control participants (28).

In a related, yet independent vein of research, the dissociative tendency for absorption and imaginative involvement in one's inner fantasy world has been specifically associated with obsessive-compulsive symptoms (17, 19, 40). A narrow attentional spotlight and heightened awareness to one's inner experience, at the expense of attention to the surroundings, may bring about uncertainty and checking (40). For example, a rigorous daily diary study on a non-clinical student sample showed that increases in absorption (specifically, immersion in internal stimuli such as one's imagination, or external stimuli such as a movie or a book) temporally preceded increases in obsessive-compulsive symptoms (19). These findings are compatible with those demonstrating the role of both dissociation and “inferential confusion” [a reliance on imagination and a distrust of the senses; (41) in predicting obsessive-compulsive symptoms in clinical and non-clinical samples (42, 43)].

The different lines of research detailed above converge to suggest possible major roles for both dissociative tendencies and obsessive-compulsive symptoms in the formation and maintenance of MD. Notably, however, while many report that their MD is experienced as compulsive, many also report that it is enjoyable, in ways that resemble addiction disorders (27, 28, 38). Thus, the daily emotions associated with MD may possibly be either negative or positive (even if consequences may be maladaptive in the long run). Because all the research on MD up to date has been cross-sectional, the identification of factors which initiate or reinforce MD is lacking. In order to advance our understanding of the nature of MD, we must employ rigorous research designs relying on a finer resolution, in which within-person hourly or daily mechanisms of change may be revealed. The present study aimed to thoroughly explore the dynamics of varying levels of MD and psychopathological distress, using a person-centered longitudinal study design, addressing change over time^4^. We explored the relations of MD with several potentially relevant psychopathological symptoms, including: dissociation, obsessive-compulsive symptoms, depression, anxiety, and social anxiety. In addition, we also explored the associations of negative and positive emotions with MD, relating to reports of MD as an addictive, gratifying behavior, which is thus difficult to control. Importantly, we set out to identify not only co-occurrence of MD with other symptoms, but also, we were interested in identifying temporal antecedents and successors of MD, in order to explicate the directional dynamics of this phenomenon.

We hypothesized that: (1) we would find concurrent relationships, meaning that days characterized by increased MD will also be characterized by increased psychopathological symptoms of other types, especially dissociative and compulsive symptoms; and that (2) we would find temporally-lagged relationships attesting to the maladaptive consequences of MD; meaning that days characterized by increased MD would be followed by an increase in symptomatology on the subsequent day. Additionally, we aimed to examine the opposite lag, meaning: which, if any, of the relevant symptoms may temporally precede increases in MD, perhaps acting as precipitating factors; this analysis was exploratory in nature, as this research is the first to conduct such an investigation. Finally, because of the addictive properties of MD, we also sought to explore concurrent and temporally-lagged relations of MD with negative and positive emotions. We hypothesized that (3) MD would be related to increases in both positive affect (due to the rewarding-addictive aspect of MD) and negative affect (due to disappointment, guilt, and shame for taking part in this activity).

It is important to underscore that the present study, unlike previous investigations in the field, focused on within-subjects, rather than between-subjects, variance. This means that we did not attempt to decipher differences between individuals, i.e., we did not ask: “are those with more severe MD prone to more severe psychopathology?”—we believe the answer to this question is affirmative, with the evidence demonstrated in previous studies reviewed above. Rather, we asked: what are the daily dynamics related to these individuals' daydreaming behavior? What types of symptoms and emotions characterize days before, during, and after they engage in intense daydreaming? Thus, we were interested in recruiting individuals who see themselves as currently suffering from MD, with varying levels of symptom severity; such variation may give rise to varying intensities across days (i.e., bouts of intense daydreaming as well as calmer periods), within individuals.

## Materials and methods

### Participants and procedure

Respondents were recruited both by calls for participation posted in online forums of MD and by email; emails were sent to individuals who have approached the second author (ES) in the past, volunteering to participate in research on MD. Individuals of consenting age, defining themselves as maladaptive daydreamers, were invited to take part in an exploratory longitudinal study on MD. Participation was voluntary with no monetary reimbursement; the participants' motivation was related to the opportunity to support rigorous scientific research on MD. Individuals who expressed an interest in participation were instructed to email a research coordinator, who, after a brief correspondence to verify their consenting age, directed them to an online informed consent form. After consenting, participants received two links, one for demographic data and trait questionnaires (which were not the focus of this study) and one for daily questionnaires, to be completed every evening for 2 weeks. Daily completions required about 15 min. The entire study was conducted online using survey software (Qualtrics, Provo, UT).

After excluding underage respondents (*n* = 4) and those not fluent in English (*n* = 1)^5^, there were 112 potential participants who emailed us with initial intent to participate in the study and were referred to the online consent form. However, seven never completed the daily questionnaires. Another participant was excluded from the daily study, because after answering the trait questionnaires she reported to the research team a host of severe confounding factors in her life which did not allow her to answer items with any certainty or accuracy. Thus, 104 started the daily study. However, some of the participants dropped out during the first few days, because of the considerable investment of time and effort that the study entailed. These were 27 participants (25.96% of those who started). Independent samples *t*-tests on age as well as on all continuous study variables at day 1 revealed no statistically significant differences between dropouts and completers, except for a slight tendency for dropouts to report higher social anxiety; however, that effect failed to reach statistical significance when performing bootstrapping, based on 1,000 resamples and bias corrected accelerated 95% confidence intervals {*M(SD)* completers = 1.92 (1.08), dropouts = 2.46 (1.21); *t*_[98]_ = 2.09, *p* = 0.04, mean difference = 0.54 [bootstrapped CI −0.06,1.18]}. We also conducted Chi square tests on dichotomous demographic variables, including gender, geographic region, whether the individual has sought professional help, and whether they had received a formal diagnosis. All were statistically non-significant except for gender, which was related to dropout; specifically, males were more likely to drop out than females [14 males among 77 completers (18.18%), vs. 11 males among 27 dropouts (40.74%); *Cramer's V* = 0.23 [bootstrapped CI: 0.04, 0.46], χ^2^_(1)_ = 5.57, *p* = 0.018].

The final sample of the daily diary study consisted of *N* = 77 (81.82% female; mean age = 29.82, *SD* = 10.18, range 18–60). Although we did not specify a criterion for the severity or maladaptation of MD required for inclusion in the study, but rather permitted individual differences in MD severity, most of our final sample (67 of the 77, which are 87%) was above the clinical cutoff score recently identified for clinical-level MD, that is 50 or above on the MDS (1). Similarly, in a study sampled in the same way (30), over 85% of the sample met this criterion; a structured clinical interview on that subset of participants demonstrated that they were highly psychopathological, with multiple co-morbid diagnoses. Almost half were unemployed and over a quarter of the sample had attempted suicide at least once. Thus, the sample of the present study comprising individuals concerned about their daydreaming, probably represents a mostly clinical sample, yet somewhat more inclusive regarding mild or moderate psychopathology. As mentioned in the introduction section, since our focus in the present study was on variance within days, we opted to include these milder cases in our sample rather than exclude them.

Participants were from 26 countries around the world; the majority (*n* = 47) were from English-speaking countries (26 from the US, 11 from the UK, 7 from Canada, and 3 from Australia). Another 13 were from European countries, 7 from Asia, 4 from Latin America, 3 from Africa and finally 3 were from the Middle East. To the question “Have you ever received psychotherapy or psychiatric help?” 53 individuals (68.80% of the sample) answered “yes.” We also asked what their presenting problem was, and whether they received a diagnosis. Forty-three supplied a diagnosis, many with several comorbidities. Frequent answers were depression (21 individuals), anxiety disorders (14 individuals), post-traumatic stress disorder or complex trauma (7 individuals), attention-deficit and hyperactivity disorder (ADHD) or attention-deficit disorder (ADD) (7 individuals), and obsessive-compulsive disorder (OCD; 5 individuals). Additional noteworthy reports were of bipolar disorder (2 individuals), borderline personality disorder (2 individuals), dissociative disorder not otherwise specified (1 individual), and psychosis (1 individual). Of those who reported that they received professional mental health aid but did not report a diagnosis, presenting problems were mostly anxiety and depression, childhood abuse, daydreaming, and compulsions. Finally, we asked “Have you been taking medications in the past 3 months? (*Yes/No*). If so, list them here.” Twenty-two individuals (28.57% of the sample) reported taking psychotropic medication. Specifically, 18 individuals were taking antidepressants or anxiolytic medication, or a combination of these [Most common were Selective Serotonin Reuptake Inhibitors (SSRIs) and Benzodiazepines]; two were taking Antipsychotics, one of them was also taking Lithium; and two were taking stimulants (Methylphenidate)^6^.

During the daily phase, participants reported MD, emotion, and psychopathology pertaining to that day, each evening before bedtime, for 14 days. They also reported other variables such as several sleep and dreaming scales, which are outside the scope of the present study. Finally, they reported the number and kind of alcoholic drinks they consumed on the previous night, if at all, in order to avoid reports of dissociative experiences that stem from the effects of intoxication. If participants skipped a night of reporting, we encouraged them to continue with the study 1 day further. The work was carried out in accordance with The Code of Ethics of the World Medical Association (Declaration of Helsinki); participants signed informed consent, and ethical considerations of this study were approved beforehand by Ben-Gurion University's institutional review board.

### Measures

#### Demographic data

Before commencing the daily study, participants reported their age, gender, country of origin, whether they had ever been to psychiatric or psychological treatment, the nature of their presenting problem, their diagnosis if they had one, and medications they may have been taking during the past 3 months.

#### Time

The time variable, spanning 14 days, was coded as 0–13 (Day 0 being the first day of the study). However, if individuals skipped a day of reporting, they would have a gap in the coding of this variable, and a higher maximum (e.g., 0–14 if they missed one night). Hence, this variable ranged from 0 to 18, although no participant had more than 14 assessments. As explained in the Supplementary Material, gaps or missing nights in the longitudinal reports tend not to compromise the statistical analyses methods used in this study.

#### Maladaptive daydreaming

The Maladaptive Daydreaming Scale [MDS; (36)] is a valid and reliable measure for the assessment of MD. Responses are given on an 11-point scale, ranging from 0% (e.g., “never,” “no urge at all”) to 100% (e.g., “very often,” “extreme urge”). For the present study, we used the original trait MDS to characterize the sample, and also adapted the MDS to a daily MD measure, with the following instructions:

In answering the following questions, please refer to your daydreaming activities today. Choose the option that best fits your experience: “0” states that the experience did not happen today, while “1” through “10” state the intensity of the experience if it did occur.

For example, item #1 was rephrased as: “I felt the need or urge to continue a daydream, that was interrupted by a real-world event, at a later point,” and item #2 as: “My daydreams were accompanied by vocal noises or facial expressions (e.g., laughing, talking, or mouthing the words),” and responses ranged from “0—not at all,” through “1—yes, very slightly” to “10—yes, extremely.” The 14 items for each day were averaged to compute a total daily *MD intensity* score. Cronbach's alpha for Day 0 was 0.92. In addition to the intensity score, we also had a *quantity* score; the quantity score was based on a single item: “Try to estimate as best as you can how many hours have you spent daydreaming in the past 24-h.” Intensity and quantity of MD represent the two outcome variables of this study.

#### Dissociation

Dissociative experiences were assessed using the Clinician Administered Dissociative States Scale (CADSS; 44), a widely-used state measure of dissociation. Specifically, we administered the 19 self-report items of the measure and did not use the optional 8-item observer-rated part. Participants were asked to report to what extent they felt different dissociative experiences “at this time” (0 = not at all, 4 = extremely). Responses were averaged to compute a total daily score. Cronbach's alpha for Day 0 was 0.90. Bremner et al. (44) reported the CADSS to have good psychometric properties.

#### Obsessive-compulsive symptoms

Obsessive-compulsive symptomatology was assessed using an adapted daily version of the items of the Obsessive-Compulsive Inventory—Revised [OCI-R; (45)]. The OCI-R is an 18-item measure assessing washing, checking/doubting, obsessing, mental neutralizing, ordering, and hoarding, using a 5-point response scale. It is widely used and has demonstrated good methodological properties. In the present study, respondents were asked to indicate to what extent each experience bothered them *today*, instead of during the past month. Items were averaged to compute a total daily obsessive-compulsive score. Cronbach's alpha for Day 0 was 0.83. This adaptation was also used in a previous daily diary study by the first author (19) and was validated in that study by demonstrating a high correlation with the trait OCI-R.

#### Depression

The assessment of daily depression was based on Nezlek and Gable (46), who administered a 3-item daily measure addressing the elements of Beck's cognitive triad (47). In the present study this measure was labeled BCT (Beck's Cognitive Triad). For any specific day during data collection, respondents reported the extent to which they felt positively about themselves, their lives, and the future, on a 7-point scale. Nezlek and Gable report that the measure exhibited good reliability and validity, validating the measure against the trait Beck depression inventory [BDI; (48)]. The items of the BCT were reversed in direction to represent negative views of the three domains and then averaged to compute a total depression score. Cronbach's alpha for Day 0 was 0.89. This measure was also used in a previous daily diary study by the first author (19) and was validated in that study by demonstrating a high correlation with trait depression using the BDI as well.

#### Anxiety

The assessment of daily anxiety was based on Marteau and Bekker (49), who recommended a shortened 6-item version of the State-Trait Anxiety Inventory [STAI; (50)], which is sensitive to fluctuations in state anxiety. Items were averaged (after correction for reverse items) to compute a total daily anxiety score. Cronbach's alpha for Day 0 was 0.88. This measure was also used in a previous daily diary study by the first author (19) and was validated in that study by demonstrating a high correlation with trait anxiety.

#### Social anxiety

Social anxiety was assessed with the Mini-SPIN (51), but asking about the past day rather than the past week. The mini-SPIN consists of three items, especially indicative of social anxiety, identified from the full 17-item Social Phobia Inventory [SPIN; (52)]. Responses are given on a 5-point scale (0 = not at all, 4 = extremely), and were averaged to compute a total daily score. The authors report good validity and reliability for this measure. In the present study, Cronbach's alpha for Day 0 was 0.81.

#### Emotion

To measure emotion, we adapted the Positive and Negative Affect Schedule [PANAS; (53)] to a daily version in the present study. The PANAS is a widely used, valid and reliable assessment tool for negative and positive emotion, by addressing several moods and feeling individuals may experience (54). In the present study, participants indicated to what extent they felt each of the 20 emotions *today*, on a 5-point Likert scale (1 = very slightly or not at all, 5 = extremely). Two scales were calculated: negative emotion and positive emotion, by averaging scores for the 10 respective emotions of each scale. Cronbach's alphas for Day 0 were 0.91 for positive emotion and 0.85 for negative emotion.

### Data analyses

First, we present partial correlations between study variables on the first day of measurements, controlling for age and gender. Next, we take into account the multiple assessment waves. The longitudinal design of the daily diary study produced a multilevel data structure (55). Consequently, multilevel linear modeling (MLM) was employed, in which level-1 daily-varying MD outcomes were predicted by level-1 daily psychopathology and emotion variables. These were nested within individuals (level-2). Thus, the relationships found in this design represent person-centered (within-subject) associations, rather than variable-centered (between-subject) associations. Multilevel modeling was implemented through SPSS mixed models (Version 23), using restricted maximum likelihood (REML) estimation, and an auto-regressive (AR1) covariance structure. Auto-regressive covariance type is appropriate for longitudinal designs, as it specifies that residuals close to each other in time with be highly correlated.

Before running the analyses, we estimated missing data and conducted multiple imputations. Details on these procedures are presented in the Supplementary Material. We also filtered out nights of heavy drinking, and details on this procedure is also covered in the Supplementary Material.

As suggested in Tabachnick and Fidell (56), “intercepts-only” models (or “null models”) were computed; These models generate two covariance parameters: a Level-1 value, representing within-subject variance (the extent to which participants vary from their own mean), and a Level-2 value, representing between-subjects variance (the extent to which participants' means vary from the general mean). From these data the intraclass correlation (ICC) may be extracted. Next, full models were specified. In each model, MD intensity or quantity was predicted by a psychopathology or emotion variable. Details on the model specification are included in the Supplementary Material. Finally, time lag analysis was employed, utilizing a 1-day lag in each direction (see Supplementary Material).

## Results

Table 1 presents partial cross-sectional correlations between all daily study variables on the first assessment wave, controlling for age and gender. As can be seen in the table, MD intensity and quantity were strongly correlated with each other, and were related to most of the distress variables. The table also presents means, standard deviations, and range for study variables, averaged for each individual across the 14 measurements. As evident from the table, on average, participants reported spending over 4 h a day daydreaming, and their average group intensity was close to the middle of the scale.

**Table 1:** Partial correlations between daily study variables on Day 0, controlling for age and gender; as well as descriptive statistics (means, standard deviations, and range) for all study variables averaged across the 14 days of the study.

	**1**	**2**	**3**	**4**	**5**	**6**	**7**	**8**	**9**
1. MD intensity	1.00								
2. MD quantity	0.67^***^	1.00							
3. CADSS	0.30^**^	0.13	1.00						
4. OCI-R	0.48^***^	0.36^**^	0.29^*^	1.00					
5. BCT	0.46^***^	0.25^*^	0.23	0.37^***^	1.00				
6. STAI	0.41^***^	0.30^*^	0.34^**^	0.45^***^	0.73^***^	1.00			
7. M.-SPIN	0.30^*^	0.23	0.53^***^	0.31^**^	0.31^**^	0.28^*^	1.00		
8. PANAS-Neg.	0.45^***^	0.31^**^	0.42^***^	0.47^***^	0.53^***^	0.57^***^	0.32^**^	1.00	
9. PANAS-Pos.	−0.28^*^	−0.09	−0.07	−0.16	−0.75^***^	−0.51^***^	−0.22	−0.21	1.00
*M*	4.80	4.03	1.35	1.39	3.89	2.23	1.76	1.71	2.20
*SD*	2.25	3.21	0.43	0.38	1.36	0.53	0.90	0.54	0.71
range	1.07, 10.01	0.07, 14.90	1.00, 3.18	1.00, 2.63	1.00, 6.83	1.10, 3.94	1.00, 4.69	1.01, 3.59	1.01, 4.44

*** *p ≤ 0.001*,

** *p ≤ 0.01*,

* *p < 0.05*.

*MD, Maladaptive Daydreaming; CADSS, Clinician-Administered Dissociative States Scale; OCI-R, Obsessive-Compulsive Inventory—Revised; BCT, Beck's Cognitive Triad; STAI, State-Trait Anxiety Inventory; M.-SPIN, Mini Social Phobia Inventory; PANAS, Positive and Negative Affect Schedule (Negative and positive scales shown separately). Statistically significant effects are italicized*.

Next, longitudinal assessments were also taken into account and for this, MLM was implemented. The “intercepts-only” models for MD intensity and quantity revealed that our MD sample had more dispersion between participants than within days (estimates for covariance parameters were 4.85 and 1.38 for between-subjects and within-subjects variance, respectively, resulting in an ICC value of 0.78 for MD intensity; Similarly, MD quantity covariance parameters were 10.09 and 2.57 for between-subjects and within-subjects, respectively, resulting in an ICC value of 0.80). However, all four of these estimates were statistically significant at the *p* < 0.001 level, suggesting that both differences between people and differences within days were varied enough to be explored, in order to find significant predictors of daily MD. The latter (differences within days) is the focus of the present investigation. The full models for contemporaneous daily relations are presented in Table 2. As can be seen in the tables, both MD outcomes were significantly related to all of the other variables, except for social anxiety which was related only to MD intensity but not MD quantity (and this is also true for anxiety, when excluding the two participants on antipsychotics). In other words, on days in which MD was more intense and time-consuming, individuals reported higher levels of dissociation, obsessive-compulsive symptoms, depression, and negative emotion, and lower levels of positive emotion. They also experienced more anxiety and social anxiety on days in which MD was more intense.

**Table 2:** Estimates of fixed effects for the psychopathology or emotion variables at Time T, contemporaneously predicting Maladaptive Daydreaming (MD) intensity or quantity at Time T.

	**Daily MD intensity**					**Daily MD quantity**				
**Parameter**	**Unstandardized estimate [CI_l_, CI_u_]**	***SE***	***t***	***p***	**Semi-partial *R*^2^**	**Unstandardized estimate [CI_l_, CI_u_]**	***SE***	***t***	***p***	**Semi-partial *R*^2^**
Dissociation	1.07 [0.64, 1.51]	0.22	4.89	< 0.001	0.10	0.78 [0.16, 1.40]	0.32	2.46	0.014	0.03
OC symptoms	1.79 [1.38, 2.21]	0.21	8.51	< 0.001	0.28	1.73 [1.11, 2.35]	0.31	5.50	< 0.001	0.12
Depression	0.33 [0.17, 0.49]	0.08	4.06	< 0.001	0.21	0.27 [0.05, 0.50]	0.12	2.37	0.018	0.08
Anxiety	0.59 [0.37, 0.81]	0.11	5.25	< 0.001	0.19	0.34 [0.01, 0.66]	0.17	2.04	0.042^$^	0.03
Social anxiety	0.34 [0.13, 0.56]	0.11	3.17	0.002	0.08	0.26 [−0.05, 0.58]	0.16	1.66	ns (0.098)	0.02
Neg. emotion	0.57 [0.33, 0.81]	0.12	4.68	< 0.001	0.14	0.36 [0.01, 0.72]	0.18	2.03	0.043	0.02
Pos. emotion	−0.43 [−0.66, −0.20]	0.12	−3.69	< 0.001	0.10	−0.34 [−0.65, −0.02]	0.16	−2.09	0.037	0.03

*Each psychopathology or emotion variable was included in a separate model, which also controlled for the time variable, the weekday/weekend variable, age, and gender. MD, Maladaptive Daydreaming; OC, obsessive-compulsive; Neg., Negative; Pos., Positive; SE, standard error; CI_l_, CI_u_, lower and upper bounds within a 95% confidence interval; ns, non-significant. All data in the table are based on the pooled results of the imputed models, except for the semi-partial R^2^, because type III tests of fixed effects are produced only for individual datasets and not for the pooled imputed results; thus, this statistic is computed based on the original data*.

$ *This effect became a statistical trend (p = 0.07) when excluding the two participants using antipsychotics*.

Next, time-lag analysis was conducted to assess whether increases in psychopathological symptoms and negative emotion, and decreases in positive emotion, preceded MD, or followed them. Thus, the analyses depicted in Table 2 were repeated, except that psychopathology or emotion predictors were now entered into the models as T–1 (Table 3, depicting psychopathology of the preceding day) or T+1 (Table 4, depicting psychopathology of the following day). As can be seen in Table 3, only obsessive-compulsive symptoms emerged as an antecedent of both MD intensity and quantity. Importantly, these effects remained statistically significant even when controlling for obsessive-compulsive symptoms at Time T; in other words, elevated obsessive-compulsive symptoms on a certain day were predictive of elevated MD on the following day, regardless of the level of obsessive-compulsive symptoms on that following day. In addition, dissociation emerged as an antecedent of MD intensity; however, this effect became statistically non-significant when including Time T dissociation in the model. As can be seen in Table 4, obsessive-compulsive symptoms, dissociation, and negative emotion emerged as successors of both MD intensity and quantity, and all of these effects remained statistically significant when including the Time T predictors in the model. This means that elevated MD on a certain day was related to increased obsessive-compulsive symptoms, dissociation, and negative emotion on the following day, over and above the contemporaneous relationship.

**Table 3:** Estimates of fixed effects for the psychopathology or emotion variables at Time T-1, predicting Maladaptive Daydreaming (MD) intensity or quantity at Time T (i.e., statistically significant effects suggest heightened psychopathology or emotion on the day preceding MD).

	**Daily MD intensity**					**Daily MD quantity**				
**Parameter**	**Unstandardized estimate [CI_l_, CI_u_]**	***SE***	***t***	***p***	**Semi-partial *R*^2^**	**Unstandardized estimate [CI_l_, CI_u_]**	***SE***	***t***	***p***	***Semi-partial R*^2^**
Dissociation	0.54 [0.10, 0.99]	0.23	2.40	0.017	0.04	0.58 [−0.02, 1.18]	0.30	1.90	ns (0.058)	0.01
OC symptoms	1.30 [0.88, 1.72]	0.22	6.03	< 0.001	0.19	1.39 [0.69, 2.09]	0.34	4.03	< 0.001	0.11
Depression	0.08 [−0.06, 0.23]	0.07	1.14	ns (>0.250)	0.02	0.06 [−0.14, 0.26]	0.10	0.59	ns (>0.250)	0.01
Anxiety	0.17 [−0.05, 0.39]	0.11	1.52	ns (0.130)	0.02	−0.02 [−0.32, 0.28]	0.15	−0.12	ns (>0.250)	0.00
Social anxiety	0.21 [−0.01, 0.42]	0.11	1.86	ns (0.063)	0.03	−0.01 [−0.31, 0.29]	0.15	−0.06	ns (>0.250)	0.00
Neg. emotion	0.10 [−0.13,0.34]	0.12	0.85	ns (>0.250)	0.01	−0.12 [−0.47, 0.23]	0.18	−0.68	ns (>0.250)	0.01
Pos. emotion	0.00 [−0.22,0.23]	0.11	0.03	ns (>0.250)	0.00	0.10 [−0.20, 0.40]	0.15	0.66	ns (>0.250)	0.00

*Each psychopathology or emotion variable was included in a separate model, which also controlled for the time variable, the weekday/weekend variable, age, and gender. MD, Maladaptive Daydreaming; OC, obsessive-compulsive; Neg., Negative; Pos., Positive; SE, standard error; CI_l_, CI_u_, lower and upper bounds within a 95% confidence interval. All data in the table are based on the pooled results of the imputed models, except for the semi-partial R^2^, because type III tests of fixed effects are produced only for individual datasets and not for the pooled imputed results; thus, this statistic is computed based on the original data*.

**Table 4:** Estimates of fixed effects for the psychopathology or emotion variables at Time T+1, predicting Maladaptive Daydreaming (MD) intensity or quantity at Time T (i.e., statistically significant effects suggest heightened psychopathology or emotion on the day following MD).

	**Daily MD intensity**					**Daily MD quantity**				
**Parameter**	**Unstandardized estimate [CI_l_, CI_u_]**	***SE***	***t***	***p***	***Semi-partial R*^2^**	**Unstandardized estimate [CI_l_, CI_u_]**	***SE***	***t***	***p***	***Semi-partial R*^2^**
Dissociation	0.78 [0.30, 1.26]	0.24	3.22	0.001	0.05	0.89 [0.18, 1.61]	0.36	2.47	0.014	0.07
OC symptoms	1.04 [0.58, 1.50]	0.23	4.45	< 0.001	0.09	1.45 [0.81, 2.08]	0.32	4.47	< 0.001	0.15
Depression	0.10 [−0.06, 0.25]	0.08	1.21	ns (0.225)	0.02	0.16 [−0.06, 0.38]	0.11	1.46	ns (0.145)	0.06
Anxiety	0.20 [−0.03, 0.44]	0.12	1.74	ns (0.082)	0.03	0.30 [−0.04, 0.63]	0.17	1.76	ns (0.080)	0.04
Social anxiety	0.22 [–0.01, 0.45]	0.12	1.89	ns (0.059)	0.05	0.17 [−0.17, 0.51]	0.17	1.00	ns (>0.250)	0.02
Neg. emotion	0.37 [0.12, 0.63]	0.13	2.91	0.004	0.05	0.40 [0.05, 0.76]	0.18	2.21	0.027	0.06
Pos. emotion	0.06 [−0.16, 0.29]	0.12	0.55	ns (>0.250)	0.00	−0.11 [−0.45, 0.23]	0.17	−0.63	ns (>0.250)	0.01

*Each psychopathology or emotion variable was included in a separate model, which also controlled for the time variable, the weekday/weekend variable, age, and gender. MD, Maladaptive Daydreaming; OC, obsessive-compulsive; Neg., Negative; Pos., Positive; SE, standard error; CI_l_, CI_u_, lower and upper bounds within a 95% confidence interval. All data in the table are based on the pooled results of the imputed models, except for the semi-partial R^2^, because type III tests of fixed effects are produced only for individual datasets and not for the pooled imputed results; thus, this statistic is computed based on the original data*.

## Discussion

Examining the characteristics of the sample, our data suggested that this was more of a clinical than a non-clinical sample; 87% were above the clinical cutoff for suspected MD, over two thirds of the sample had been in therapy at least once in their lives, and over half of the sample reported having received at least one psychiatric diagnosis. This finding is in accordance with previous research suggesting that MD is characterized by high levels of concomitant psychopathology (30). On average, as a group, on their first assessment participants reported spending 4.5 h actively engaging in daydreaming on that single day, suggesting that their MD was indeed time-consuming and excessive, taking up over a quarter of their waking time. Even though all of our participants defined themselves as suffering to some extent from MD, the sample was heterogeneous in the amount and intensity of their MD. In addition to this heterogeneity, participants varied in their own daily intensity and quantity of MD during the period of the study, which enabled us to explore patterns of co-variation with daily changes in psychopathological symptoms and emotion.

Our exploration of temporal covariation between the variables demonstrated strong contemporaneous associations; in other words, on evenings in which individuals reported more intense and time-consuming MD, they also reported elevated levels of a host of other psychopathological symptoms, as well as increased negative, and decreased positive, emotion for that day. The contemporaneous relationships were non-specific and spanned the full range of psychopathological symptoms included in the study. These findings are in accordance with our first hypothesis and strengthen the participants' own definition of their daydreaming as maladaptive. Although individuals with MD commonly report their daydreaming to be pleasurable and addictive (26), in the present study we found no evidence for an increase in positive emotion associated with increased MD. In line with subjective reports by individuals who engage in this mental activity (35), the positive effects of MD activity are probably more short-term and may be detected in an experience-sampling study, tapping into experience at any present moment. Our daily diary study, in which symptoms were reported in the evening to describe the day that had passed, showed that overall, days characterized by increased MD were characterized by negative emotion. Perhaps when reflecting upon the day, individuals felt shame and regret for the time wasted in daydreaming.

Negative emotion was also a successor of MD; it was elevated on the following day, even when controlling for the contemporaneous relation (i.e., for the same day's negative emotion). Although our data cannot directly distinguish between *cause* and *course*, they show that an elevation in MD was followed by an increase in negative emotion, strengthening the notion that in the long term (i.e., on the next day), MD does not promote pleasurable sensations but rather results in psychological distress (even if in the shorter term, it may be characterized by increased pleasure, which was not detectible by our design). Such a lagged effect was also found for state dissociation symptoms (using a measure that addresses mostly depersonalization and derealization) and obsessive-compulsive symptoms: they were elevated on the day following MD. These findings partially support our second and third hypotheses and add to them by demonstrating specificity of the effects to dissociative and obsessive-compulsive psychopathologies, and negative, rather than positive, emotion. We will first discuss dissociation, and then obsessive-compulsive symptoms.

Possibly, engaging in daydreaming for several hours compromises the sense of presence in reality and brings about experiences of depersonalization and derealization, because attention had been focused on internal fantasy rather than external stimuli and reactions to those stimuli. Indeed, focusing attention outwards by sensory grounding techniques has been found useful in aiding dissociative individuals (57, 58). Moreover, in MD, not only is attention focused inwards, but it is focused on fantasized (thus, derealized) “characters,” performing activities and engaging in their own dialogues (27). Possibly, attending to mental imagery which is attributed to a non-self entity (i.e., a “character”), produces impairment in one's normal sense of embodiment. Indeed, increased bodily sensations may be characteristic of intensified daydreams (59). Interestingly, patients with depersonalization-derealization disorder (DPD) were as good as controls in detecting their own heartbeat, despite their core complaint of disembodiment (60); the authors of that study suggested that the impairment in DPD is not perceptual but rather, that DPD individuals have a difficulty in integrating the percepts into their sense of self. We suggest that engaging in fantasy about non-self or idealized-self entities may bring about disembodiment and difficulty in integration of the self, represented by depersonalization. The temporal relationship between daydreaming and dissociation in this time-lag study conducted on adult respondents, raises questions about the developmental trajectories and temporal relationship between immersive childhood daydreaming under adverse conditions and possible subsequent development of dissociative symptoms. Further studies are needed in order to better understand the temporal relations between intensified daydream imagery and dissociative symptomatology, in general, and a sense of embodiment, in particular.

Notably, although dissociation seems to be a central feature of MD, it did not precede MD. This may be due to our 1-day lag, which is perhaps not the ideal lag to detect such an effect. Conversely, it may stem from the type of dissociative experience measured; possibly, MD may be a form of pathological absorption. In other words, MD may be instigated from an absorptive dissociative tendency to lose oneself in one's imagination, while depersonalization-derealization may be merely a consequence of MD. It is also possible that MD does not stem from dissociation but is a type of dissociative symptom in itself (i.e., there is no causal relation, but rather, they are one and the same).

As mentioned above, obsessive-compulsive symptoms were also elevated in the days following MD; In addition, they were the only construct in this study which consistently *preceded* MD. Notably, despite the finding that obsessive-compulsive symptoms are a central mechanism in the daily dynamics of MD, only a small subset of our sample reported having a diagnosis of OCD (5 participants, which amounts to only 6.49% of the sample). This discrepancy suggests that obsessive-compulsive symptoms and MD share common mechanisms and interact with each other on one hand, but MD does not seem to be merely a subtype of OCD on the other hand [also see (30)]. Many individuals with MD report that they are constantly drawn to daydreaming in a compulsory fashion, and have difficulty controlling their thoughts (26, 35). Future studies should clarify whether MD is similar to an obsession (as in thoughts and images which appear uninvited and are experienced as intrusive), or a mental compulsion (as in a mental action that one feels an urge to perform).

Although MD may be construed as a type of obsession or mental compulsion in itself, this construal cannot fully account for the findings of this study. Notably, the measure used to assess obsessive-compulsive symptoms in this study focuses mainly on various types of specific compulsions, such as checking, counting and washing, with only a few items focusing on obsessing or mental lack of control^7^. The elevation of these symptoms before, as well as after, an elevation in MD, in a vicious cycle of compulsions, points to shared mechanisms between the two disorders; for example, perhaps low levels of Serotonin may be involved in the development and maintenance of MD, and should thus be targeted for pharmaceutical interventions. Indeed, as reviewed in the introduction, one case study of MD reported successfully treating the patient with SSRIs (39). Importantly, compulsions in OCD are reported to alleviate anxiety and distress (61), which reinforces their occurrence; however, in the present study anxiety did not emerge as a predecessor of MD. This lack of finding may stem from methodological issues; specifically, our design, as mentioned above, was a daily-diary design rather than an experience-sampling design, and the occurrence of anxiety before MD may be grounded in subtler lags (e.g., a few minutes, rather than 1 day). It is yet to be explored in future studies whether MD is elevated following more proximal increases in anxiety. Alternatively, this lack of effect may represent a reality, which distinguishes MD from OCD. It is also noteworthy that whereas the majority of compulsions in OCD mostly function to alleviate anxiety, mental compulsions are different; individuals with OCD reported that they performed them mostly in an automatic manner, without an explicit reason (61).

The relationship between obsessive-compulsive symptoms and automatic behavior is relevant to the discussion on associations between OCD and dissociation. It has been claimed that dissociative absorption, which includes acting on “auto-pilot,” brings about uncertainty for one's actions and therefore may instigate repeated checking, and that on the other hand, repetition brings about depersonalization and derealization, together creating a dissociation-OCD cycle (40). Support for the role of absorption in strengthening or predicting future obsessive-compulsive symptoms has been found in two longitudinal studies on non-clinical samples (17, 19). There is also support for the role of repetition or perseveration, a symptom of OCD, in creating derealization and dissociative-like vagueness of memory (62, 63), and this effect is more prominent in individuals suffering from OCD (64). Indeed, OCD seems to be characterized by an excessive reliance on imagination [“inferential confusion”; (41)] and by feelings of detachment from the world (65). Thus, it seems that the relationship between obsessive-compulsive symptoms and dissociation is a two-way streak, in which one reinforces the other. Similarly, in the present study elevations in obsessive-compulsive symptoms both preceded, and followed, elevations in MD, suggesting a vicious cycle in which engaging in one's internal imagery and performing compulsions strengthen each other.

The present study raises an important question regarding MD: is MD a separate phenomenon, a psychopathological disorder in its own right, or merely a symptom of another disorder? And if it is the latter, is MD a subtype of dissociation, OCD, or something else entirely? The findings raise the possibility of construing MD as a type of obsession or mental compulsion, however, obsessions in OCD are usually related to feelings of intrusion and anxiety, whereas MD is described as more voluntary and enjoyable. Evidence shows that MD is experienced as a highly rewarding behavior marked by behavioral addictive characteristics (27–29, 36, 38). Indeed, it has been proposed that in chronically addicted individuals, maladaptive behaviors and high relapse rates may be better conceptualized as being “compulsive” in nature as a result of dysfunction within inhibitory brain circuitry (66). Future research should also distinguish between MD and Attention Deficit Hyperactivity Disorder (ADHD), which are highly comorbid (30). Inattention is disruptive to functioning, and similarly, mind-wandering has been shown to be related to distress (67). Although we believe these constructs are not identical to MD, because inattention and mind-wandering do not include fanciful daydreaming, more research on MD is needed in order to attempt to distinguish MD from related constructs.

Finally, the findings of the present study may aid in illuminating implications for possible psychotherapeutic interventions useful for treating MD. The close relationship of MD with obsessive-compulsive and addictive symptomatology may indicate the potential usefulness of cognitive-behavioral approaches in the form of response prevention [inspired by Exposure and Response Prevention, ERP; e.g., (68)], in attempting to curb MD. Indeed, a recent pioneering case study attempting to formulate a treatment plan for MD, utilized ERP-informed interventions, among others (20). For example, MD was avoided and intercepted by changing the endings of plots to aversive ones. That study also incorporated mindfulness training (69) into the treatment; such training has been shown to be useful for treating obsessive-compulsive symptoms (70, 71) and has been hypothesized to be useful for dissociation as well, as the notion of staying in the present moment is somewhat opposite to retreating to dissociative states (72). It is too early to determine the usefulness of these interventions for the treatment of MD, as their impact should be examined in randomized and controlled clinical trials; however, the findings of the present study provide support for their theoretical rationale in the context of MD.

Limitations of the study should be noted. First, the study was conducted online, using solely self-report measures, which may be biased and suffer from shared method variance. Future studies should use the recently-developed SCIMD (1) in order to diagnose participants with standard criteria applied by clinicians rather than self-diagnoses. The issue of relying solely on self-report rather than a clinician's interview is especially important considering that the sample seems to be highly psychopathological (as is evident from the percentage of suicidality and unemployment). However, individual differences in the tendency to exaggerate symptoms probably did not substantially influence the results of this study, which were person-centered rather than variable-centered^8^. Second, because of the exploratory nature of this study (due to the scarcity of research in the field of MD), criteria for inclusion in the study sample were expansive; participants were of a wide age range and resided in several countries. Despite the overall high rates of psychopathology, our sample also probably included various levels of psychopathological distress. Nevertheless, as in the previous limitation, the heterogeneity of the sample is also less problematic when considering that this study focused on within-subject longitudinal dynamics. In addition, the inclusion of different ages and countries of origin is also an advantage because results may be generalized to diverse MD populations. Another demographic which is of importance is gender; the sample comprised mostly women, possibly influencing the results; however, this may be representative of the MD population. For example, in an earlier study, 83% of individuals seeking online peer support for MD were reported to be female (26). Furthermore, female overrepresentation is characteristic of psychiatric samples in general, especially vis-à-vis internalizing—rather than externalizing—mental health issues (74). Notably, however, even within the scope of individuals with MD, males were more likely to drop out of this study; although we controlled for gender in all analyses, it is possible that our results are more representative of MD dynamics for females. Moreover, our sample represents individuals who agreed to devote considerable time and effort for the scientific advancement of the MD field; thus, self-selection may have influenced our findings and generalization should be approached with caution. For example, the relation between MD and negative emotion may have been influenced by the sample, i.e., individuals who view their daydreaming as maladaptive. Finally, another source of sampling bias may have affected generalizability: the study's language and online medium excluded populations of non-English speakers, the poorer and less educated strata of society and those who are not internet-savvy (e.g., the elderly). Replication studies sampling from more diverse populations could render further support to our findings.

Nevertheless, the present study has several strengths of scientific rigor; it is the first longitudinal exploration of MD, relying on experience-near daily diaries instead of retrospective self-report, thus reducing susceptibility to bias. It rests on a diverse international clinical sample; although we did not conduct psychiatric assessments, we view this sample as a primarily clinical one, not only based on their MDS scores, but also because they reportedly suffer from daydreams that interfere with their lives and cause significant distress, and the majority of these individuals have sought professional help for their symptoms. The study spanned 14 days with repeated assessments and utilized advanced statistical analyses methods (including time lag analysis) to show which variables increase before, and which increase after, the daily increases in MD, which may aid us in understanding the dynamics of this phenomenon. Our findings strongly support the notion that these individuals' daydreaming is indeed maladaptive, as it is accompanied, as well as followed by, increases in psychopathological symptoms and negative emotion. Additionally, the finding that a surge in obsessive-compulsive symptoms precedes MD points to a key role of this construct as a contributing mechanism. We hope that our findings will aid future attempts to develop therapy guidelines for individuals battling MD, so that they will be able to take control over their compulsion to daydream.

## Data availability statement

The datasets for this manuscript are not publicly available because in the consent form signed by participants, it was stated that the data are confidential and will be available only to the researchers, NS and ES, for the purpose of statistical analysis. Requests to access the datasets should be directed to Dr. Nirit Soffer-Dudek: soffern@bgu.ac.il.

## Author contributions

NS-D is responsible for study design and conceptualization, sample recruitment, data collection and analyses, literature search, drafting the manuscript, and subsequent revisions and editing. ES is responsible for study design and conceptualization, sample recruitment, literature search, revisions, and editing.

### Conflict of interest statement

The authors declare that the research was conducted in the absence of any commercial or financial relationships that could be construed as a potential conflict of interest.

## Footnotes

^1^Assessment measures for FP such as the Inventory of Childhood Memories and Imaginings [ICMI; (20, 23)] and the Creative Experiences Questionnaire [CEQ; (24)] include childhood experiences that are not necessarily indicative of normal daydreaming, for example, belief in fictional characters as real ones, loneliness, and feeling that you are someone else, as well as items tapping adult experiences that also assess domains which do not represent a propensity for normal daydreaming, like belief in paranormal experiences.

^2^Maladaptive Daydreamers forum (n.d.). Available online at: https://groups.yahoo.com/neo/groups/maladaptivedaydreamers/info (Accessed February 21, 2017).

^3^Wild Minds Network (n.d.). Available online at: http://wildminds.ning.com/ (Accessed February 21, 2017).

^4^As opposed to variable-centered analyses, which simply focus on the relationships between variables, person-centered analyses are those which estimate how variables group within individuals, tapping into a different source of variance.

^5^Based on the email correspondence with the research coordinator, for which he used internet-based translation software, it was clear that this individual's skill level in English would not allow him to properly understand the items.

^6^Study results were unchanged when excluding the two participants who reported use of antipsychotics (one of them was the participant reporting a diagnosis of psychosis), unless stated otherwise.

^7^Associations between obsessive-compulsive symptoms and MD remained even when removing such items from the total score.

^8^A fixed effect in a mixed model is the effect of X on Y within each grouping unit, averaged across grouping units (73), which in this case is individuals. Any stable personality trait of symptom exaggeration should not have affected daily relationships between psychopathology and MD, as it would remain constant within each individual. However, daily relations could have been inflated if individuals tended to mark higher scores on all questionnaires in certain days, because of shared method of assessment.

## Abbreviations

DPD: Depersonalization-Derealization Disorder
FP: Fantasy Proneness
MD: Maladaptive Daydreaming
SCIMD: Structured Clinical Interview for Maladaptive Daydreaming.
