# Supplementary material for: Trapped in a Daydream: Daily Elevations in Maladaptive Daydreaming Are Associated With Daily Psychopathological Symptoms
Source: Front Psychiatry. 2018 May 15;9:194. doi: 10.3389/fpsyt.2018.00194 (PMC5962718; doi:10.3389/fpsyt.2018.00194)
Supplement: Supplementary file 1 [file Data_Sheet_1.DOCX]

Supplementary Material

Trapped in a Daydream: Daily Elevations in Maladaptive Daydreaming Are Associated with Daily Psychopathological Symptoms

Nirit Soffer-Dudek*, Eli Somer

*** Correspondence:** Nirit Soffer-Dudek: soffern@bgu.ac.il

# Supplementary information for data analyses section

Missing data estimation revealed that 7 participants had less than 14 assessments (each had between 11 and 13), resulting in 1,067 samples rather than 1,078 (98.98%). Notably, MLM models are efficient in handling gaps and missing assessment waves well, with no need for missing data completion (1). Statistical assumptions are not compromised; only power is slightly reduced by the missing assessments (which were, in any case, only ~1%). However, variables skipped on assessment waves that did take place, are important to consider and deal with. We estimated this type of missingness patterns using the missing values analysis function of SPSS (version 23). Little’s MCAR test was statistically significant (*χ^2^*_[23]_ = 38.45, *p* = .02), but separate *t*-tests meant to explore if missingness is random or not could not be computed because missingness was slightly lesser than 3%. Missingness lesser than 5% means that non-response is probably ignorable and any method of dealing with it will probably yield the same results (1). Nevertheless, we employed multiple imputations (2, 3), which are considered by many to be the “gold standard” for managing missing data. All analyses were conducted on the imputed dataset, and pooled estimates are reported throughout the paper.

Next, we sought to filter out nights of heavy drinking. On most nights (973, which are 91.19%) participants reported no alcohol consumption.^[[1]](#footnote-1)^ Their reports of the amount and type of alcoholic beverages consumed on the previous night were coded using a standard drink calculator, according to which, a standard drink is one that contains 0.6 fluid ounces (14 grams) of pure alcohol.^[[2]](#footnote-2)^ For women, heavy drinking is considered to be 4 standard drinks or more, although for men the standard is higher, specifically, 5 or more.^[[3]](#footnote-3)^ We relied on the more stringent criterion of 4 and above (especially considering that most of our sample were women), which resulted in filtering out 25 nights (which represent 0.02% of the overall sampled nights, or 26.60% of the nights in which drinking occurred).

The main models were specified so that daily MD was predicted by other daily symptoms or emotion. The variable representing time was coded with values 0–13 and included as a predictor in each model in order to de-trend it. In addition to time, each model controlled for age, gender, and a dichotomous variable indicating whether this was a weekday or a rest day (i.e., Saturday and Sunday).^[[4]](#footnote-4)^ We controlled for this variable because being at work or at home may be related to MD patterns or to levels of distress. All continuous predictors were grand-mean centered (see 4 on the importance of centering, as well as 5 who support grand-mean over group-mean centering for a multilevel model with no interactions). Age and gender were specified as fixed effects, whereas time, the weekday/weekend variable, and the variable of interest (psychopathology or emotion) were specified both as fixed and as random effects. This means that the predictors’ intercepts and slopes were allowed to vary among individuals. For each effect of psychopathology or emotion, a standardized effect size of explained variance was calculated, namely *semi-partial R^2^* (6). Importantly, predictors were not all included in one omnibus model because psychopathological symptoms tend to increase and decrease together across days (7). Multicollinearity is even more problematic in multilevel models than in ordinary regression (1).

For time-lag analysis models, psychopathology or emotion variables were entered into the model as T-1 (e.g., Day 1 psychopathology predicts Day 2 MD) or T+1 (e.g., Day 3 psychopathology predicts Day 2 MD), to explore directionality of effects (see 8). Note that these analyses have somewhat lower power than concurrent analyses, as only 13, rather than 14, measurements are used. Finally, a more rigorous exploration of temporality assessed whether these effects remained statistically significant even when including the predictor at time T in the model (for example, Day 1 psychopathology predicts Day 2 MD, even when controlling for Day 2 psychopathology). This suggests a temporal effect which cannot be explained by contemporaneous relationships (also see 8).

**References**

1 Tabachnick BG, Fidell LS. *Using multivariate statistics (5th ed.).* Boston, MA: Allyn and Bacon. (2007). 1008 p.

2 Rubin DB. *Multiple Imputation for Nonresponse in Surveys.* New York: J. Wiley & Sons. (1987). 258 p.

3 Schafer JL, Olsen MK. Multiple imputation for multivariate missing-data problems: A data analyst's perspective. *Multivariate Behav Res* (1998) 33(4):545-71.

4 Cheng J, Edwards LJ, Maldonado-Molina MM, Komro KA, Muller KE. Real longitudinal data analysis for real people: Building a good enough mixed model. *Stat Med* (2010) 29(4):504-20.

5 Wu YWB, Wooldridge PJ. The Impact of Centering First-Level Predictors on Individual and Contextual Effects in Multilevel Data Analysis. *Nurs Res* (2005) 54(3):212–6.

6 Edwards LJ, Muller KE, Wolfinger RD, Qaqish BF, Schabenberger O. An R^2^ Statistic for Fixed Effects in the Linear Mixed Model. *Stat Med* (2008) 27(29):6137–57.

7 Soffer-Dudek N. Daily elevations in dissociative absorption and depersonalization in a non-clinical sample are related to daily stress and psychopathological symptoms. *Psychiatry* (2017) 80(3):265-78*.* doi: 10.1080/00332747.2016.1247622

8 Killingsworth MA, Gilbert DT. A wandering mind is an unhappy mind. *Science* (2010) 330(6006):932.

1. This means that the drink variable was highly skewed, and thus was more suitable to use as a filter variable, than to control for within the regression models. [↑](#footnote-ref-1)
2. Thinking about drinking (n.d.a). Available at: <http://thinkingaboutdrinking.msu.edu/index.php?option=com_content&view=article&id=3&Itemid=13/> (accessed 14 January 2016). [↑](#footnote-ref-2)
3. Thinking about drinking (n.d.b). Retrieved from <http://thinkingaboutdrinking.msu.edu/index.php?option=com_content&view=article&id=6&Itemid=16/> (accessed 8 November 2016). [↑](#footnote-ref-3)
4. Two participants were from Israel, in which the weekend rest days are Friday and Saturday, whereas Sunday is a weekday. Thus, the dichotomous variable was coded accordingly for these participants. [↑](#footnote-ref-4)
